# Polymeric Nanogels for Antimicrobial Therapy

**DOI:** 10.3390/gels12030264

**Published:** 2026-03-22

**Authors:** M. Cristina Ibarra-Alonso, Sofía Estrada-Flores, Alejandra E. Herrera-Alonso, Elsa Nadia Aguilera-González, Antonia Martínez-Luévanos

**Affiliations:** 1 SECIHTI-Facultad de Ciencias Químicas, Universidad Autónoma de Coahuila, Saltillo 25280, Coahuila, Mexico; ibarra.cristina@uadec.edu.mx; 2 Facultad de Ciencias Químicas, Universidad Autónoma de Coahuila, Saltillo 25280, Coahuila, Mexico; sofiaestrada@uadec.edu.mx (S.E.-F.); alejandra.08ha@gmail.com (A.E.H.-A.); elsaaguilera@uadec.edu.mx (E.N.A.-G.)

**Keywords:** antimicrobial therapy, nanogel structure, nanogel synthesis, polymers

## Abstract

At present, the development of antimicrobial systems requires ongoing and consistent improvement in their efficacy and versatility. Polymeric nanogels can serve as an efficient tool for this purpose, as they have become an excellent alternative for the design of tissue engineering and bone regeneration scaffolds, in addition to vehicles for the delivery of drugs or active substances, and they have recently been investigated as wound dressings. Nanogels have also been shown to be an excellent alternative for nanomedicine due to their antimicrobial activity and specific properties, such as swelling, biocompatibility, and biodegradability. In this review, we present an analysis of the use of polymeric nanogels for antimicrobial therapy and provide a discussion focused on different types of nanogels and their advantages and disadvantages, which will serve as a reference point for the future development of nanogels with antimicrobial properties. We also focus on the analysis of the different methodologies employed to prepare nanogels.

## 1. Introduction

To combat the various difficulties faced in the management of bacterial infections caused by resistant microorganisms, several methodologies for the generation of new effective compounds in antimicrobial therapy have been developed [1]. The efficient delivery of antibiotics or antimicrobial active substances to the site of infection is a promising strategy in antimicrobial therapy. The size of polymeric nanogels is an advantage in antimicrobial therapy as it enables them to interact at the cellular level and effectively combat pathogenic organisms [2].

Nanogels are nanometer-sized hydrogels with similar properties, possessing a high capacity to retain large amounts of water—up to a thousand times their dry weight [3]. In addition, their highly porous structure makes them excellent carriers for the delivery of metal nanoparticles, antibiotics, and other antimicrobial substances.

In this study, we report relevant findings on nanogels used in antimicrobial therapy, outlining their strengths and limitations, supporting their use in the development of future nanogel-type polymeric materials for effective antimicrobial therapy.

## 2. Relevance of Polymeric Nanogels for Antimicrobial Therapy

Polymeric nanogels are nano-sized, crosslinked, inflatable hydrogel network structures. Hydrogels can be formed by various polymers, for example, natural (collagen, fibrin, alginate, chitosan (CS), hyaluronic acid, and polypeptides, among others), synthetic (polyacrylamide, polyethylene glycol, polyvinyl alcohol, and polyacrylamide), or hybrids, which are a combination of synthetic and natural polymers [4]. They have a wide range of applications, with respect to their physical and chemical properties, depending on their chemical composition, type of charge, degradability kinetics, and porosity, among others [5]. Nanogels are also mostly biocompatible, and like hydrogels, they are sensitive to external stimuli, making them potential candidates for various applications, such as tissue engineering, vehicles for drug dosing, or antimicrobial agents in wound dressings and prostheses; they are also employed in the cosmetics industry and agricultural sector [6,7]. The antibacterial efficacy of nanogels arises from several distinct mechanisms. For instance, the inherent negative charge of bacterial membranes facilitates the application of positively charged nanogels, such as those synthesized from CS, through electrostatic interactions. Furthermore, nanogels serve as effective delivery vehicles for metallic ions (e.g., Ag^+^) or natural bioactive compounds such as thymol. The controlled release of these therapeutic agents can be achieved by engineering stimuli-responsive nanogels designed to discharge their payload in response to specific triggers, such as an acidic pH environment [8]. The nanometric size of these systems is responsible for their interaction with bacteria and other pathogenic microorganisms occurring at the cellular level. The size of bacteria varies from 0.2 nm to 700 nm, depending on their shape, i.e., rod (bacillus), spherical (coccus), or spiral (spirillum) [9]. Rod-shaped bacteria are between 0.5 μm and 4 μm in width and less than 15 μm in length; in comparison, coconut-shaped bacteria possess an average diameter of around 0.5 to 1.0 μm [9,10].

The relevance of polymeric nanogels for use in antimicrobial therapy lies in their nanometer size, surface functionalization, and ability to load or deliver antimicrobial drugs, substances, or particles, and they are currently the focus of extensive research worldwide. In this review, research on polymeric nanogels for antimicrobial therapy is discussed [11,12,13,14,15,16].

### 2.1. Overview of Polymer Nanogels

Nanogels are described by Martínez et al., as aqueous dispersions of nanometer-sized polymeric particles formed after physical or chemical crosslinking procedures [17]. The three-dimensional nanoscale lattices of these polymeric nanogels range from 100 to 200 nm; they can be physically or chemically crosslinked across different functional groups, such as hydroxyl, carboxyl, sulfonic, and amino groups [18,19,20,21]. Nanogels possess excellent properties such as biocompatibility, stability, high load-bearing capacity, modifiable surfaces, and adjustable size [20,21].

### 2.2. Nanogel Synthesis Methods

**Figure 1:**
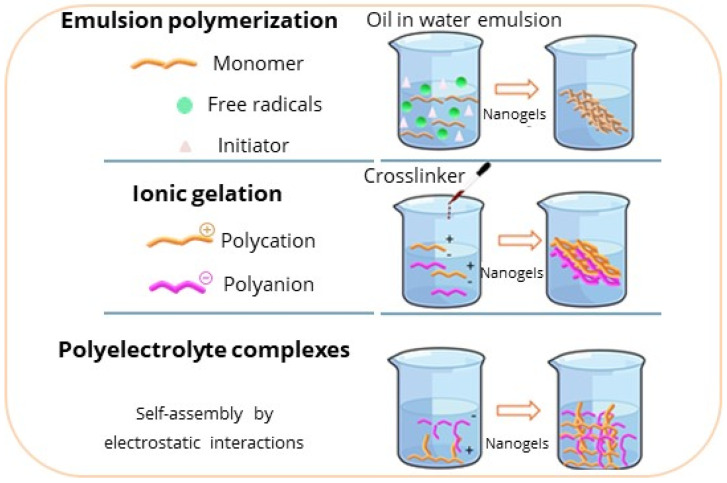
Scheme showing different synthesis methods for nanogel preparation.

Different methods can be employed for the synthesis of nanogels, for example, emulsion crosslinking, microemulsions, mini-emulsions (water in oil), and, depending on the initial size of the dispersed aqueous phase, other methods are also employed, such as the formation of reverse micelles, ionic gelation, or the mechanism of self-assembly through the construction of polyelectrolyte complexes [2], as presented in Figure 1. Through these methods, various parameters and properties can be controlled, such as the size and degree of swelling, using surfactants, polymer concentration, polarity of the molecules, pH, and functional groups.

#### 2.2.1. Emulsion

The emulsion method is a heterogeneous polymerization technique. It is performed in environments with an oil-in-water phase (direct micelles) or water-in-oil (reverse micelles) [22]. In a typical oil-in-water reaction, radicals are generated from the breakdown of chemical primers. Radicals react with other monomers, and consequently, the chain grows. One of the disadvantages of emulsion polymerization is the use of organic solvents [23]; however, increasing research attention is directed toward the development of environmentally suitable polymerization methods [23,24].

Ding et al., developed CS-based nanogels, preparing a single and double emulsion, and incorporated the systems into an alginate hydrogel as a microcapsule matrix for the delivery of probiotics to the gastrointestinal tract [23]. They performed multilayer encapsulation of probiotics, which are encapsulated in the innermost layer of a single or double emulsion, and the outer layer consists of alginate, which provides greater biocompatibility.

Sui et al., obtained poly (2-diisopropylaminoethyl methacrylate) (PDPA) nanogels through emulsion polymerization, testing them with different surfactants (sodium dodecylsulfate (SDS), Cetyltrimethylammonium Bromide (CTAB), Poloxamer P 123, and Pluronic F127) [24]. In addition, they investigated the size variation based on the reaction time and the amount of monomer added. From their results, they found that the nucleation rate and growth rate are dependent on the added monomer concentration. The authors note that they obtained smaller sizes and greater nanogel stability by using nonionic surfactants, such as poloxamer P 123, because the micelles of the nonionic surfactants could capture free radicals from the initiated oligomers at a faster rate; as a result, the size of the nanogels increased with the increasing amount of crosslinking in this case. Additionally, surfactant-free emulsion polymerization is a commonly used technique for obtaining nanogels with monodispersed particle size distributions. This method is considered an environmentally friendly option. However, one of the limitations of this method is its slow polymerization rate and resulting poor stability. When an emulsion is formed without the use of surfactants, the water-insoluble polymer chains in the core are predicted to collapse and form the nanogel precursors with a slow rate of polymerization to form the nanogel, as shown in Figure 1.

#### 2.2.2. Ionic Gelation

The ionic gelation method is based on the ability of polyelectrolytes to crosslink in the presence of counterions. The polyelectrolytes dissociate in polar solvents, resulting in charges in the polymer chains and the release of counterions in the solution, and through the addition of another polyelectrolyte of a different charge to this solution, ionic gelation occurs. Nanogels can be formed through covalent bonds or physical crosslinking, such as hydrogen bonds and hydrophobic or electrostatic interactions. These interactions lead to ionic gelation and precipitates, resulting in the formation of spherical particles, as shown in Figure 1 [25,26,27]. Some notable examples of polyelectrolytes are CS, hyaluronic acid, alginate, cellulose, collagen, and poly-acrylic acid.

Shitrit & Bianco-Peled, obtained pectin-and CS-based hydrogels through physical crosslinking with tripolyphosphate (TPP) at pH 5.5 and chemical crosslinking with genipin [27]. The hydrogels were stable under acidic conditions and size control was achieved. Genipin was then added to achieve covalent crosslinking. They noted that at pH 5.5, the size of the nanogels increased with increasing CS concentration.

Zhang et al., developed keratin-crosslinked sodium alginate nanogels with the ionic gelation method using the NH_2_ groups of keratin, combined with the COOH and OH groups of the alginate, resulting in an ionic gelation process whereby keratin acts as an ionic crosslinker (Figure 1) [28]. The authors focused on the influence of alginate concentration on nanogel thickness.

Algharib et al., synthesized CS and carboxymethyl CS-based nanogels through ionic gelation and electrostatic interactions between the positive charges of CS and the anionic part of carboxymethyl CS [29]. They used TPP as a crosslinking agent to achieve greater stabilization in the structure. They obtained spheres of roughly 171 nm in size loaded with rifaximin, which exhibits broad-spectrum antibacterial activity against anaerobic and aerobic bacteria, both Gram-positive and Gram-negative.

#### 2.2.3. Developing a Polyelectrolytic Complex

Polyelectrolyte complexes arise from self-assembly interactions, driven by entropy between the polyelectrolyte and another macromolecule or molecule of opposite charge, forming electrostatic complexes. This self-assembly process enables the fabrication of nanogels, as shown in Figure 1 [30]. Polyelectrolyte complexes are versatile formulations based on macromolecules with ionic or ionizable groups (polyanions and polycations).

Rusu et al., obtained nanogels based on polysaccharides such as proteins, polypeptides, or natural polyelectrolytes by developing polyelectrolyte complexes [31]. The authors reported that polysaccharides can form complex polyelectrolytes in aqueous solutions and are stabilized by secondary and electrostatic forces (hydrogen bonds and hydrophobic interactions). For example, the formation of protein complexes and polysaccharides. Their findings highlight the formation of nanogels based on CS functionalized with maleic anhydride (MAC) and proteins, such as bovine serum albumin (BSA), through a self-assembly technique, resulting in an ecological, simple, and low-cost process. The nanogels were characterized, and their antibacterial properties after being loaded with amoxicillin (Amox)—a broad-spectrum antibiotic—were investigated.

Bhattacharjee et al., developed a system of nanogels based on CS/poly methacrylic acid (CS/PMMA), taking advantage of CS’s cationic nature and ability to be chemically coupled with anionic macromolecules to form complex polyelectrolytes (Figure 1) [32]. The formation of the complex between CS and PMMA was achieved by secondary forces. The authors obtained nanogels of roughly 200 nm in size.

In antibacterial therapy, ionic gelation and polyelectrolyte complexation are the most commonly applied methods due to their simplicity and avoidance of harmful chemicals; furthermore, polyelectrolytes possess intrinsic antibacterial properties through membrane disruption and electrostatic interactions with bacterial cells [33].

The selection of an appropriate synthesis strategy is a critical factor in determining the physicochemical properties and biological performance of nanogels. While various techniques have been developed to tailor these systems for antimicrobial delivery, each method presents a unique set of technical advantages and inherent limitations. To provide a clear comparative perspective, in Table 1, we summarize the fundamental mechanisms and primary strengths and weaknesses of the most prominent synthesis routes currently employed in antibacterial research.

### 2.3. Morphology and Structure of Nanogels for Antimicrobial Therapy

**Figure 2:**
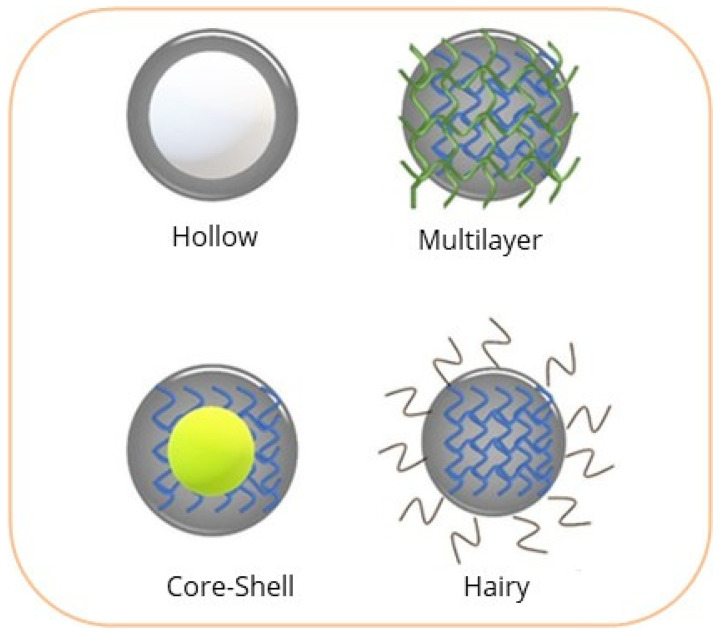
Main structures of nanogels.

In antimicrobial therapy, the morphology and structure of nanogels are of great importance for incorporating host molecules. Antimicrobial efficacy is enhanced by combining the structural benefits of hydrogels with a nanometric size, which facilitates the effective incorporation of diverse host molecules. An antimicrobial agent can contain several types of host molecules, e.g., metal nanoparticles [11,12], antibiotics [34,35], and active substances [36,37]. The effective incorporation of the host molecule and its subsequent release will depend directly on its structure. Shah et al., [10] and Preman et al., [35], classified nanogels into four main types of structures: hollow nanogel, multilayer nanogel, core–shell nanogel, and hairy nanogel. Figure 2 schematically presents the different structures of a nanogel.

#### 2.3.1. Hollow Nanogels

Hollow nanogels are characterized by a hollow interior, providing a cavity that can be used for the loading of drugs, active substances, or metal nanoparticles. They are primarily obtained by crosslinking polymers into an inorganic particle, which is then removed to provide the structure of hollow nanogels [38,39]. For example, poly(N-isopropylacrylamide) (PNIPAM) with silica, as noted by Rudov et al., yields a micro/nanogel in the form of “hollow nanogels” using silica particles in the core as a sacrificial template, after the introduction of the appropriate solvent; as a result, a cavity is formed in the center, with PNIPAM serving as the thermosensitive outer layer [38]. In addition, Zafaryab et al., developed temperature-responsive hollow nanogels with silica as a template [40]. Xing et al. fabricated hollow nanogels with an interpenetrating polymer network structure based on poly(acrylic acid) (PAA) and PNIPAM with a stimuli response to pH and temperature to load isoniazid in the cavities of the hollow nanogel [41].

#### 2.3.2. Multilayer Nanogels

Multilayer nanogels are mainly composed of multiple layers of either a single polymer or multiple polymers. The selection of polymers plays an important role in forming this nanogel structure. The self-assembly method is ideal for preparing nanogels in multilayers [42,43,44], involving the sequential deposition of cationic and anionic polymers to form thin layers fabricated in the nanoscale range in a simple manner, with reproducible thicknesses and adjustable compositions [44]. Zavgorodnya et al., developed multilayer nanogels of poly(N-vinylcaprolactam) (mPVCL)—a heat-sensitive polymer—by crosslinking acrylic acid in the final layer [42]. Lancellotti et al., developed a polymeric coating of polyethylene glycol (PEG) with antibacterial properties, resulting from the release of minocycline covalently bound to polyethylene glycol [43].

#### 2.3.3. Core–Shell Nanogels

Nanogels with a core–shell morphology can possess a metallic core [12], ceramic [38,45,46] or polymeric [47], and a polymeric shell [45,46,47], with a spherical-type morphology, which are the most common in recent studies and consist of a spherical central particle, completely covered by a different material. This type of core–shell morphology provides effective control of either antibiotic, active substance, or metallic nanoparticle release, resulting in effective materials for use in antimicrobial treatment [10,11,34,35,36]. Rezvani Ghomi et al., developed nanogels with a core–shell structure, in which titanium oxide (TiO_2_) modified with 3-(trimethoxysilyl)propyl methacrylate (MPS) formed the nucleus; the shell is formed by a copolymer of acrylamide (AAm) and 2-acrylamide-2-methyl propane sulfonic acid (AMPS), resulting in a highly hydrophobic nanogel, due to its composition [45]. In the case of polymer–polymer core–shell hydrogels, the ionic gelation method is the most commonly employed technique to synthesize this type of nanogel with a core–shell structure. Ionic gelation refers to, as its name suggests, the gelation and crosslinking of a polyelectrolyte in the presence of counterions [48]. Hesan et al., fabricated nanogels with a core-coated structure as drug delivery substrates using alginate biopolymers as a core surrounded by a CS layer for the loading and release of an antibiotic [47].

#### 2.3.4. Hairy Nanogels

Hairy nanogels primarily arise from copolymers joined by covalent bonds or grafted polymers, although they can also take the form of polymer chains linked through physical interactions (hydrogen bonding), hydrophobic interactions, and electrostatic interactions. These nanogels possess a linear chain linked to several side chains [49,50,51,52,53,54]. These side chains give rise to hairy or comb-like strands or structures, which extend to the surface, interacting with the contact medium. Hairy nanogels are formed by crosslinking the networks, either chemically and/or physically, with brush-shaped strands [50,51]. Yu et al., assembled comb-type nanogels based on butyl acrylate, glycidyl methacrylate, and hexafluorobutyl methacrylate, generating a homogeneous comb-like nanogel that is covalently bonded [52]. Emulsion polymerization, following the RAFT [53] and Pickering [54] methods, is the most commonly employed synthesis technique to obtain hairy nanogels.

In Table 2, we provide a comparative overview of how these distinct nanogel morphologies influence the loading, release profiles, and overall antibacterial efficiency of the resulting delivery systems.

### 2.4. Smart or Stimuli-Sensitive Nanogels

**Figure 3:**
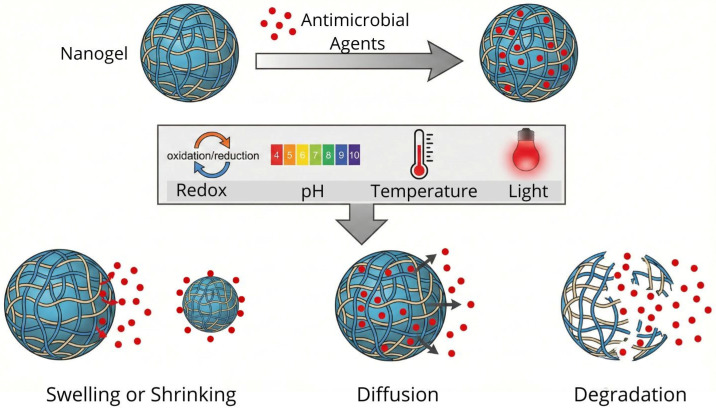
Schematic illustration of the nanogel in response to different stimuli.

A significant advantage of nanogels is their capacity to respond to environmental triggers—such as changes in pH [35,54,55,56,57], temperature [58,59,60], light [61,62], or redox potential [63,64]—depending on the properties of their polymeric matrix. The stimuli facilitate the controlled release of antimicrobial agents through diffusion or matrix degradation, establishing these systems as highly promising delivery vehicles (Figure 3).

#### 2.4.1. pH-Responsive Nanogels

pH influences swelling or shrinking behavior. CS exhibits poor solubility in an alkaline environment and tends to contract; in contrast, in an acidic pH, the crosslinked structure tends to expand due to the protonation of the amino group. Bhattacharjee et al., synthesized nanogels using the pH-sensitive CS/PMAA-based ionic gelation method; these nanogels show great potential for use in antimicrobial therapy [32]. Wang et al., developed CS-based nanogels loaded with Tanshinone, an active substance that can improve antibacterial efficacy against *Streptococcus mutans* (*S. mutans*) [36]. The CS coating not only protected the active substance from harsh environments, due to its pH response, but also selectively released it under acidic conditions. Another example of pH-sensitive nanogels is those based on hyaluronic acid. Yang et al., designed nanogels based on hyaluronic acid methoxy-poly (ethylene glycol) and diethylentriamine that respond to pH, due to the formation of a benzoic imine bond [55].

#### 2.4.2. Temperature-Responsive Nanogels

Thermosensitive nanogels expand as the temperature changes. A representative polymer sensitive to temperature changes is PNIPAM. Nanogels made of PNIPAM expand in response to the low temperature of the solution due to the hydrogens of the solution and the amides of the PNIPAM repelling each other at low temperatures, and conversely, they exhibit a hydrophobic state when the temperature of the solution increases and contraction occurs in the nanogels [58,59,60,61].

Kim et al., developed copolymerized PNIPAM hollow nanogels with acrylic acid contents with thermosensitive and pH-sensitive properties [60]. In addition, Yurdasiper et al., prepared PNIPAM nanogels loaded with triclosan, and through thermosensitivity analysis, evaluated their antibacterial efficacy on *Cutibacterium acnes* [61]. PNIPAM-based nanogels can replace bacterial topical medications, as their nanometer size overcomes skin barriers and improves dermal penetration, avoiding the side effects of conventional medications, which mainly irritate the skin and are associated with other serious effects.

#### 2.4.3. Redox-Responsive Nanogels

Nanogels are sensitive to redox potential, which is related to changes in pH and oxygen content, and the change in redox potential can be exploited as a trigger stimulus in the release of a certain antimicrobial agent. The disulfide bonds/bridges incorporated into polymeric nanogels impart them with the ability to be stimulated by a redox environment; therefore, the nanogels are endowed with disulfide bonds, which are susceptible to reduction [63,64,65]. Notabi et al., conducted a study on a nanogel of diethylaminoethyl methacrylate-co-hydroxyethyl methacrylate-g-polyethylene glycol monomethacrylate (DEAEMA-co-HEMA-g-PEGMA), crosslinked with a disulfide crosslinker (bis(2-methacryloyloxyethyl)). They reported that the dissociation of this nanogel is mediated by reduced glutathione (GSH), a mechanism that effectively facilitates the controlled release of antimicrobial agents [63]. Laradji et al., developed nanogels based on hyaluronic acid, cystamine, and cholesterol, with a redox response, by crosslinking a thiol-disulfide, which is released in a reducing environment [64].

#### 2.4.4. Light-Responsive Nanogels

In this class of nanogels, the integration of light-cleavable moieties or plasmonic nanomaterials—such as gold nanorods and silver nanoparticles—enables a dual-action therapeutic approach. Upon exposure to near-infrared (NIR) irradiation, these systems undergo photothermal heating, which facilitates the eradication of embedded bacteria through protein denaturation and membrane rupture. Furthermore, this photothermal effect promotes biofilm disassembly and the cleavage of sensitive chemical bonds, thereby triggering the controlled release of encapsulated antimicrobial payloads [18].

Innovative light-triggered systems have been developed in recent years to enhance the temporal control of antimicrobial delivery. For instance, Ballesteros et al., fabricated polycaprolactone (PCL) nanofiber mats functionalized with photoresponsive chitosan nanogels containing silver nanoparticles [66]. This hybrid platform utilizes light activation to modulate the release of active substances, thereby providing a tunable mechanism to suppress bacterial growth. Similarly, Sun et al., designed photoresponsive nanogels using a hyaluronic acid framework to encapsulate ciprofloxacin [67]. Their findings demonstrated that light irradiation significantly enhanced the inhibitory efficacy of the loaded nanogels against both Staphylococcus aureus and Salmonella typhimurium, highlighting the potential of light-mediated therapies in overcoming conventional drug limitations.

### 2.5. Antimicrobial Agents Incorporated into Nanogels

Many of the advantages of nanogels are associated with their nanometer size, with them being able to penetrate the cell wall of bacteria and subsequently release the antimicrobial agent, whether metal nanoparticles [11,12,68], active substances [36,38], or antibiotics [47,48,49].

#### 2.5.1. Metal Nanoparticles

**Figure 4:**
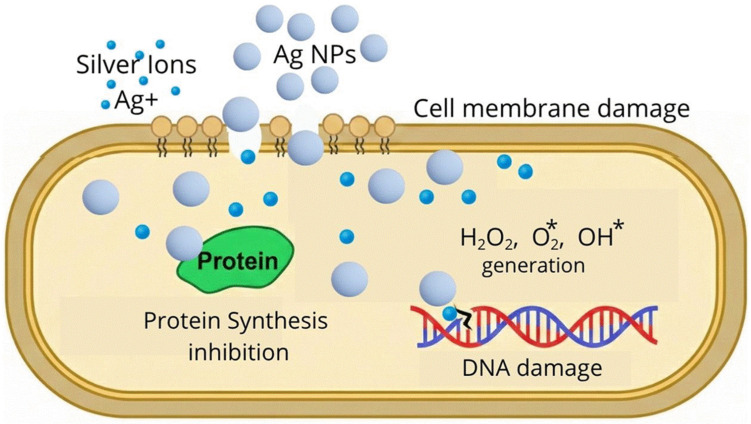
Mechanism of action of nanoparticles against bacteria.

The incorporation of nanometer-sized metal particles, such as silver (Ag), gold (Au), and copper (Cu) nanoparticles, into polymeric nanogels has emerged as a strategy for antimicrobial therapy. This approach is primarily driven by the potent antimicrobial properties of these metals, which offer an effective alternative for combating persistent bacterial infections [11,12,68]. Silver nanoparticles (Ag-NPs) are the most widely employed as they exhibit strong bactericidal activity against drug-resistant bacteria [68]. Ag-NPs are a source of silver ions (Ag^+^) and play a vital role in antimicrobial activity, as, due to the positive charge of these ions, they can form complexes with nucleic acids, in addition to exhibiting electrostatic attractions and affinity towards sulfur proteins, enabling silver Ag^+^ ions to adhere to the cytoplasm and cell wall. As soon as the cells absorb the free Ag^+^ ions, the respiratory enzymes are deactivated, leading to the production of reactive oxygen species (ROS), for example, OH^*^, O_2_^*^, and H_2_O_2_, resulting in the disruption of adenosine triphosphate (ATP) release, leading to cell apoptosis; this mechanism of antimicrobial action is identical to that exhibited by other metal nanoparticles [69]. The mechanism of action of nanoparticles against bacteria is described in Figure 4.

**Figure 5:**
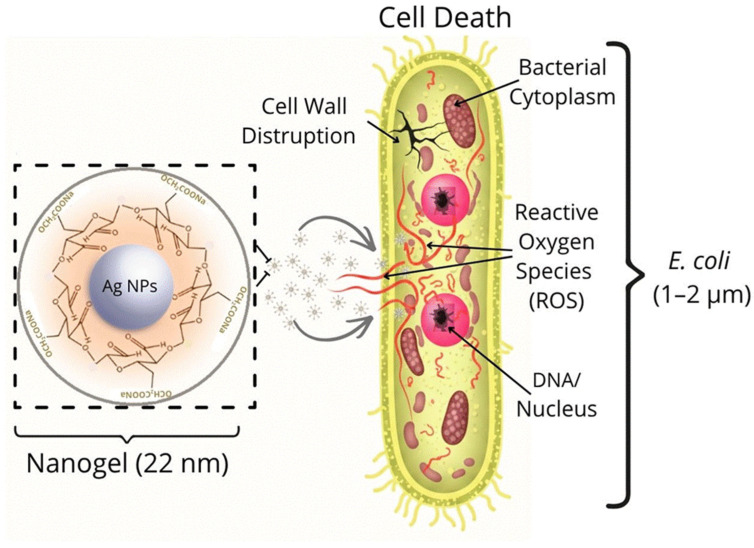
Nanogel based on oxidized carboxymethylcellulose and nanosilver. Images illustrating its mechanism of cell wall disruption and its induction of oxidative stress in bacteria (ROS).

Gao et al., employed the reverse microemulsion technique to prepare nanogels composed of CS/Ag, achieving effective antibacterial activity and low cytotoxicity [12]. Somani et al. prepared a nanogel of 22 nm in size, with a core–shell structure based on oxidized carboxymethylcellulose and nanosilver, demonstrating significant antimicrobial activity against *E. coli* and *S. aureus* bacteria [13]. Its effectiveness lies in the fact that the nanogel altered the bacterial cell wall, generating reactive oxygen species within the cell, which caused cell death (Figure 5). The use of silver nanoparticles in polymeric nanogels provides an excellent option for antimicrobial therapy.

In this context, metal oxide nanoparticles, such as metal nanoparticles, are an attractive alternative to combat microbes that are highly resistant to various classes of antibiotics. The different properties of metal oxides contribute to an efficient antimicrobial response; for example, the photocatalytic and semiconductor properties of titanium dioxide (TiO_2_) nanoparticles could be exploited to inhibit the growth of bacteria [70,71]. Other types of oxides, such as zinc oxide (ZnO) [72], cuprous oxide (Cu_2_O) [73], and magnesium oxide (MgO) [74], could be effectively used for this purpose. However, it is important to note that the antimicrobial efficacy of these metal oxides primarily lies in the nanometer size of the particles [70].

#### 2.5.2. Active Substances

Moghadam et al., used oil extracted from lemon peel as an active substance [37]. This lemon essential oil was loaded into a nanogel made of gelatin and guar gum, resulting in an inhibitory effect on the growth of *E. coli* and *S. aureus* bacteria.

**Figure 6:**
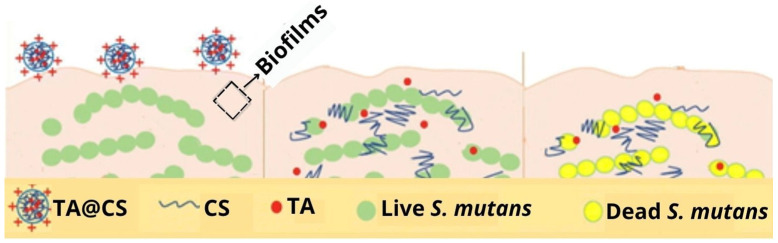
Scheme showing how encapsulated CS nanogels from Tanshinone IIA (TA@CS) penetrate the biofilm and release TA, killing the bacterium *S. mutan*. Adapted from reference [36].

Tanshinone IIA, which could be considered an active substance, is derived from the dried roots of Salvia miltiorrhiza, a Chinese herb; it has anti-inflammatory and cardioprotective properties, in addition to antimicrobial properties [75]. Wang et al., developed CS-based nanogels to encapsulate Tanshinone IIA (TA), to improve antibacterial efficiency against *Streptococcus mutans* (*S. mutan*) (Figure 6), benefiting the food and pharmaceutical industries, in addition to other sectors [36].

**Figure 7:**
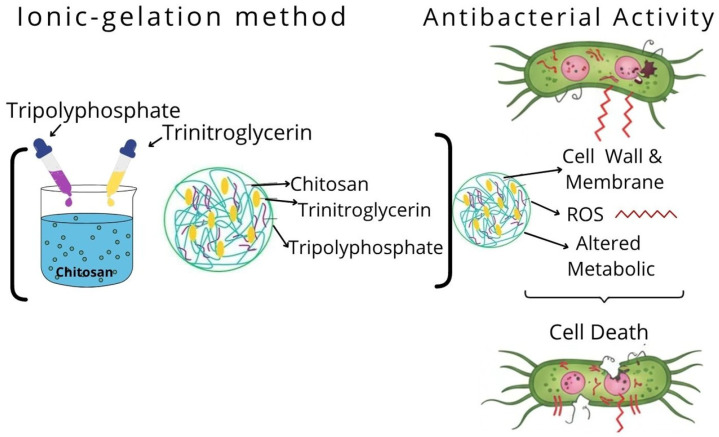
Scheme of CS nanogels encapsulating trinitroglycerin and its antimicrobial activity.

Asadi et al., synthesized CS nanogels with TPP, using the ionic gelation technique, loaded with trinitroglycerin (TNG) [76]. The resulting nanogels exhibited antimicrobial activity against *S. aureus*, *E. coli*, methicillin-resistant *S. aureus*, and vancomycin-resistant *Enterococci*. Figure 7 presents a diagram of the synthesis of CS nanogels, the encapsulation of TNG, and its release.

Conversely, TNG is a nitric oxide (NO)-releasing agent. NO exhibits antibacterial properties by binding to the surface of bacteria, altering crucial bacterial components, such as the cell wall and membranes, inhibiting metabolic activity, and generating reactive oxygen species (ROS). However, NO has an extremely short half-life, which limits its diffusion and adequate dose; the synthesis of TNG-loaded nanogels therefore increases the bioavailability of NO, in addition to providing sustained release and targeted delivery [76]. From these results, we can conclude that active substances represent an invaluable source of antimicrobial compounds with enormous therapeutic potential.

#### 2.5.3. Antibiotics

The objective of incorporating an antibiotic into nanogels is to promote its availability in the biological environment and prevent bacterial proliferation through its antimicrobial mechanism, which consists of inhibiting the synthesis of the bacterial cell wall. Antibiotics also inhibit deoxyribonucleic acid (DNA) replication and protein synthesis metabolism [67], while remaining toxic to surrounding tissue and reducing the effective dose of antibiotics loaded into nanogels [47,48,77]. Some current examples of the use of antibiotics in nanogels for antibacterial therapy are described below.

**Figure 8:**
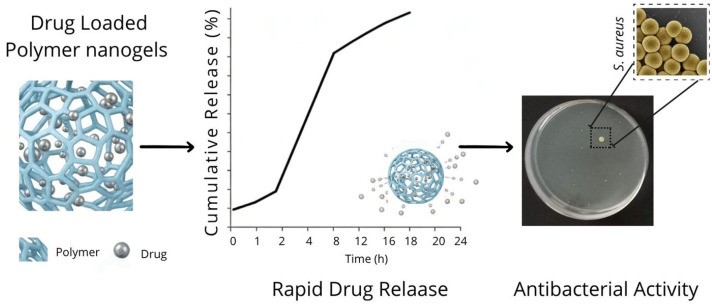
Drug-loaded crosslinked nanogels.

Hesan et al., utilized mupirocin, loaded in alginate and CS-based nanogels featuring a core–shell structure [47]. While these antibiotics exhibit broad-spectrum efficacy against Gram-positive bacteria, this research group aimed to address their inherent deficiency in inhibiting Gram-negative strains through this nano-formulation. Mupirocin is frequently used in the treatment of superficial skin infections caused by *S. aureus*. Vancomycin is an efficient antibiotic against Gram-positive and Gram-negative bacteria. Zhang et al., loaded vancomycin into CS-coated borate and hydroxyapatite microspheres [46]. Vancomycin provided highly effective antibacterial activity against *S. aureus* and *E. coli*. Saracoglua et al., synthesized polyethylene glycol digylcidyl ether (PEGDE)-loaded starch nanogels to inhibit the growth of *E. coli* and *S. aureus*, with a hydrodynamic diameter around 100 nm and a degradation time of 120 min, which showed great promise for antimicrobial therapy (Figure 8) [77].

## 3. Concluding Remarks

In this review, we present recent advances in nanogels and their application in antimicrobial therapy. The size of polymeric nanogels provides an advantage in antimicrobial therapy, primarily by facilitating interactions with cells or their targeted delivery at the cellular level to fight pathogenic organisms. The efficient delivery of antibiotics, metal nanoparticles, or active substances to the site of infection in a precise manner is a strategy currently exploited in antimicrobial therapy. The morphology or structure of nanogels plays a crucial role, as their ability to incorporate a host molecule, which confers antimicrobial properties, depends on the type of morphology. In particular, nanogels with a core–shell structure are the most widely employed type at present, compared to other types of structures (holes, hair, and multilayer). The most widely researched polymers in the production of core–shell nanogels are CS, alginate, and a copolymer comprising polyacrylamides and polymethacrylate. Core–shell nanogels are used in antimicrobial therapy for the loading and release of drugs, e.g., vancomycin and mupirocin, which are used in the treatment of skin infections. These nanogels possess good antimicrobial properties. In this context, active substances represent an invaluable source of antimicrobial agents; for example, substances such as Tanshinone IIA, derived from dried sage roots and oil extracted from lemon peel, are currently recognized antimicrobial agents, resulting in an inhibitory effect against the bacteria *E. coli* and *S. aureus*. The use of metal nanoparticles and metal oxides incorporated into polymeric nanogels as a promising strategy against bacterial infections was also discussed, mainly due to their antimicrobial effect. It is also important to note that certain nanogels exhibit distinct advantages over others, as, depending on their polymeric matrix, they respond to certain stimuli such as changes in pH, temperature, light, and redox, with them being widely used for the efficient release of antimicrobial agents.

Ionic gelation techniques, the formation of polyelectrolytic complexes, and emulsion polymerization have gained increasing popularity in recent years because they enable the development of nanogels. It is worth noting that researchers around the world are focusing their efforts on producing bacterial nanogels, mainly due to their nanometric size, as an alternative against microbes resistant to various types of antibiotics.

## Figures and Tables

**Table 1:** Comparative analysis of nanogel synthesis routes, mechanisms, key advantages, major limitations, and large-scale potential.

Method	Mechanism	Key Advantages	Major Limitations	Large-Scale Potential	References
Emulsion polymerization	Polymerization in oil/water	High control over the size of the nanogel	Slow rate of polymerization	High	Gao et al. (2022) [12], Keskin et al. (2021) [2], Lekjinda et al. (2023) [22]
Ionic gelation	Physical crosslinking of polyelectrolytes	Aqueous-based synthesis and simple and versatile	Ionic strength changes	Moderate to high	Fasiku et al. (2022) [11], Kyzioł et al. (2022) [25]. Gadziński et al. (2023) [34]
Polyelectrolyte complexes	Self-assembly driven by entropy and electrostatic interactions between oppositely charged macromolecules	Solvent-free, simple, and cost-effective synthesis	Highly dependent on the charge density of precursors	Moderate	Ding et al. (2023) [23], Kwame et al. (2023) [30], Rusu et al. (2020) [31]

**Table 2:** Comparative analysis of nanogel morphologies in antimicrobial therapy.

Morphology	Loading Capacity	Release Profile	Antibacterial Efficiency	References
Hollow	Very high because of the cavity acting as a reservoir	Permeability could be controlled if there is a stimuli-sensitive shell	High, for a rapid release of the loading	Rudov et al. (2023) [38], Xing et al. (2021) [6]
Core–shell	High, drugs can be loaded both in the core or in the shell matrix	Sustained, the shell could act as a diffusion barrier	Prolonged, the barrier allows specific bacterial targeting	Rezvani et al. (2023) [45], Zhang et al. (2023) [46], Ding et al. (2023) [49]
Multilayer	Moderate because of dependence on the number of layers, where the loading can be distributed	With degradation of the layers, this process can also be controlled if there is a responsive system	The loading can be released in stages	Zavgorodnya et al. (2017) [42], Lancelloti et al. (2023) [43], Alotaibi et al. (2023) [44]
Hairy	Moderate loading would not be effective for the entrapment of the drug with the crosslinked network	Diffusion of the drug for rapid interaction between the brushes and the medium	Brushes can provide contact killing of the bacterial membrane	Yang et al. (2017) [50], Uhlik et al. (2022) [51], Yu et al. (2023) [52], Fu et al. (2017) [53]

## Data Availability

No new data were created or analyzed in this study.
